# Application of Nanotechnology in Food Science: Perception and Overview

**DOI:** 10.3389/fmicb.2017.01501

**Published:** 2017-08-07

**Authors:** Trepti Singh, Shruti Shukla, Pradeep Kumar, Verinder Wahla, Vivek K. Bajpai, Irfan A. Rather

**Affiliations:** ^1^Department of Microbiology, Gurukula Kangri University Haridwar, India; ^2^Department of Energy and Materials Engineering, Dongguk University-Seoul Seoul, South Korea; ^3^Department of Forestry, North Eastern Regional Institute of Science and Technology Itanagar, India; ^4^Department of Applied Microbiology and Biotechnology, Yeungnam University Gyeongsan-si, South Korea

**Keywords:** nanoparticles, food safety, food preservation, functional food, food nutrition, nano-processed food products

## Abstract

Recent innovations in nanotechnology have transformed a number of scientific and industrial areas including the food industry. Applications of nanotechnology have emerged with increasing need of nanoparticle uses in various fields of food science and food microbiology, including food processing, food packaging, functional food development, food safety, detection of foodborne pathogens, and shelf-life extension of food and/or food products. This review summarizes the potential of nanoparticles for their uses in the food industry in order to provide consumers a safe and contamination free food and to ensure the consumer acceptability of the food with enhanced functional properties. Aspects of application of nanotechnology in relation to increasing in food nutrition and organoleptic properties of foods have also been discussed briefly along with a few insights on safety issues and regulatory concerns on nano-processed food products.

## Introduction

Over the past few decades, nanotechnology has increasingly been considered as to be attractive technology that has revolutionized the food sector. It is a technology on the nanometer scale and deals with the atoms, molecules, or the macromolecules with the size of approximately 1–100 nm to create and use materials that have novel properties. The created nanomaterials possess one or more external dimensions, or an internal structure, on the scale from 1 to 100 nm that allowed the observation and manipulation of matter at the nanoscale. It is observed that these materials have unique properties unlike their macroscale counterparts due to the high surface to volume ratio and other novel physiochemical properties like color, solubility, strength, diffusivity, toxicity, magnetic, optical, thermodynamic, etc. (Rai et al., 2009; Gupta et al., 2016). Nanotechnology has brought new industrial revolution and both developed and developing countries are interested in investing more in this technology (Qureshi et al., 2012). Therefore, nanotechnology offers a wide range of opportunities for the development and application of structures, materials, or system with new properties in various areas like agriculture, food, and medicine, etc.

The rising consumer concerns about food quality and health benefits are impelling the researchers to find the way that can enhance food quality while disturbing least the nutritional value of the product. The demand of nanoparticle-based materials has been increased in the food industry as many of them contain essential elements and also found to be non-toxic (Roselli et al., 2003). They have been also found to be stable at high temperature and pressures (Sawai, 2003). Nanotechnology offers complete food solutions from food manufacturing, processing to packaging. Nanomaterials bring about a great difference not only in the food quality and safety but also in health benefits that food delivers. Many organizations, researchers, and industries are coming up with novel techniques, methods, and products that have a direct application of nanotechnology in food science (Dasgupta et al., 2015).

The applications of nanotechnology in food sector can be summarized in two main groups that are food nanostructured ingredients and food nanosensing. Food nanostructured ingredients encompass a wide area from food processing to food packaging. In food processing, theses nanostructures can be used as food additives, carriers for smart delivery of nutrients, anti-caking agents, antimicrobial agents, fillers for improving mechanical strength and durability of the packaging material, etc. whereas food nanosensing can be applied to achieve better food quality and safety evaluation (Ezhilarasi et al., 2013). In this review, we have summarized the role of nanotechnology in food science and food microbiology and also discussed some negative facts associated with this technology.

## Nanotechnology in Food Processing

The nanostructured food ingredients are being developed with the claims that they offer improved taste, texture, and consistency (Cientifica Report, 2006). Nanotechnology increasing the shelf-life of different kinds of food materials and also help brought down the extent of wastage of food due to microbial infestation (Pradhan et al., 2015). Nowadays nanocarriers are being utilized as delivery systems to carry food additives in food products without disturbing their basic morphology. Particle size may directly affect the delivery of any bioactive compound to various sites within the body as it was noticed that in some cell lines, only submicron nanoparticles can be absorbed efficiently but not the larger size micro-particles (Ezhilarasi et al., 2013). An ideal delivery system is supposed to have following properties: (i) able to deliver the active compound precisely at the target place (ii) ensure availability at a target time and specific rate, and (iii) efficient to maintain active compounds at suitable levels for long periods of time (in storage condition). Nanotechnology being applied in the formation of encapsulation, emulsions, biopolymer matrices, simple solutions, and association colloids offers efficient delivery systems with all the above-mentioned qualities. Nano polymers are trying to replace conventional materials in food packaging. Nanosensors can be used to prove the presence of contaminants, mycotoxins, and microorganisms in food (Bratovčić, 2015).

Nanoparticles have better properties for encapsulation and release efficiency than traditional encapsulation systems. Nanoencapsulations mask odors or tastes, control interactions of active ingredients with the food matrix, control the release of the active agents, ensure availability at a target time and specific rate, and protect them from moisture, heat (Ubbink and Kruger, 2006), chemical, or biological degradation during processing, storage, and utilization, and also exhibit compatibility with other compounds in the system (Weiss et al., 2006). Moreover, these delivery systems possess the ability to penetrate deeply into tissues due to their smaller size and thus allow efficient delivery of active compounds to target sites in the body (Lamprecht et al., 2004). Various synthetic and natural polymer-based encapsulating delivery systems have been elaborated for the improved bioavailability and preservation of the active food components (**Table 1**). Further, the importance of nanotechnology in food processing can be evaluated by considering its role in the improvement of food products in terms of (i) food texture, (ii) food appearance, (iii) food taste, (iv) nutritional value of the food, and (v) food shelf-life. It is a matter of fact that surprisingly nanotechnology not only touches all the above-mentioned aspects but has also brought about significant alterations in food products providing them novel qualities.

**Table 1:** Different nanotechniques to encapsulate and delivery of functional ingredients.

Nanotechnique	Characteristic feature	Examples	Reference
Edible coatings	To preserve the quality of fresh foods during extended storage	Gelatin-based edible coatings containing cellulose nanocrystal	Fakhouri et al., 2014
		Chitosan/nanosilica coatings	Shi et al., 2013
		Chitosan film with nano-SiO_2_	Yu et al., 2012
		Alginate/lysozyme nanolaminate coatings	Medeiros et al., 2014
Hydrogels	Can be easily placed into capsules, protects drugs from extreme environments, and to deliver them in response to environmental stimuli such as pH and temperature	Protein hydrogels	Qui and Park, 2001
Polymeric micelles	Solubilize water-insoluble compounds in the hydrophobic interior, high solubility, low toxicity	PEO-b-PCL [poly(ethylene glycol)block-poly(caprolactone)] polymeric micelles	Ma et al., 2008
		Methoxy poly(ethylene glycol) palmitate polymeric micelles	Sahu et al., 2008
Nanoemulsions	(i) Greater stability to droplet aggregation and gravitational separation;	β-Carotene-based nanoemulsion	Kong et al., 2011
	(ii) Higher optical clarity; and, (iii) increased oral bioavailability	β-Carotene-based nanoemulsion	Yuan et al., 2008
Liposomes	Since liposome surrounds an aqueous solution inside a hydrophobic membrane, it can be used delivery vehicles for hydrophobic molecules (contained within the bilayer) or hydrophilic molecules (contained in the aqueous interior)	Cationic lipid incorporated liposomes modified with an acid-labile polymer hyper-branched poly(glycidol) (HPG)	Yoshizaki et al., 2014
Inorganic NPs	They display good encapsulation capability and their rigid surfaces allow controlled functionalization	Mesoporous silica nanoparticles	Tang et al., 2012


### Texture, Taste, and Appearance of Food

Nanotechnology provides a range of options to improve the food quality and also helps in enhancing food taste. Nanoencapsulation techniques have been used broadly to improve the flavor release and retention and to deliver culinary balance (Nakagawa, 2014). Zhang et al. (2014) used the nanoencapsulation for the highly reactive and unstable plant pigment anthocyanins which have various biological activities. Through, encapsulating cyanidin-3-O-glucoside (C3G) molecules within the inner cavity of apo recombinant soybean seed H-2 subunit ferritin (rH-2) improved the thermal stability and photostability. This design and fabrication of multifunctional nanocarriers for bioactive molecule protection and delivery. Rutin is a common dietary flavonoid with great important pharmacological activities but due to poor solubility, its application in the food industry is limited. The ferritin nanocages encapsulation enhanced the solubility, thermal and UV radiation stability of ferritin trapped rutin as compared to free rutin (Yang et al., 2015). The use of nanoemulsions to deliver lipid-soluble bioactive compounds is much popular since they can be produced using natural food ingredients using easy production methods, and may be designed to enhance water-dispersion and bioavailability (Ozturk et al., 2015).

As compared to larger particles which generally release encapsulated compounds more slowly and over longer time periods, nanoparticles provide promising means of improving the bioavailability of nutraceutical compounds due to their subcellular size leading to a higher drug bioavailability. Many metallic oxides such as titanium dioxide and silicon dioxide (SiO_2_) have conventionally been used as color or flow agents in food items (Ottaway, 2010). SiO_2_ nanomaterials are also one of the most used food nanomaterials as carriers of fragrances or flavors in food products (Dekkers et al., 2011).

### Nutritional Value

A majority of bioactive compounds such as lipids, proteins, carbohydrates, and vitamins are sensitive to high acidic environment and enzyme activity of the stomach and duodenum. Encapsulation of these bioactive compounds not only enables them to resist such adverse conditions but also allows them to assimilate readily in food products, which is quite hard to achieve in non-capsulated form due to low water-solubility of these bioactive compounds. Nanoparticles-based tiny edible capsules with the aim to improve delivery of medicines, vitamins or fragile micronutrients in the daily foods are being created to provide significant health benefits (Yan and Gilbert, 2004; Koo et al., 2005). The nanocomposite, nano-emulsification, and nanostructuration are the different techniques which have been applied to encapsulate the substances in miniature forms to more effectively deliver nutrients like protein and antioxidants for precisely targeted nutritional and health benefits. Polymeric nanoparticles are found to be suitable for the encapsulation of bioactive compounds (e.g., flavonoids and vitamins) to protect and transport bioactive compounds to target functions (Langer and Peppas, 2003).

### Preservation or Shelf-Life

In functional foods where bioactive component often gets degraded and eventually led to inactivation due to the hostile environment, nanoencapsulation of these bioactive components extends the shelf-life of food products by slowing down the degradation processes or prevents degradation until the product is delivered at the target site. Moreover, the edible nano-coatings on various food materials could provide a barrier to moisture and gas exchange and deliver colors, flavors, antioxidants, enzymes, and anti-browning agents and could also increase the shelf-life of manufactured foods, even after the packaging is opened (Renton, 2006; Weiss et al., 2006). Encapsulating functional components within the droplets often enables a slowdown of chemical degradation processes by engineering the properties of the interfacial layer surrounding them. For example, curcumin the most active and least stable bioactive component of turmeric (*Curcuma longa*) showed reduced antioxidant activity and found to be stable to pasteurization and at different ionic strength upon encapsulation (Sari et al., 2015).

## Nanotechnology in Food Packaging

A desirable packaging material must have gas and moisture permeability combined with strength and biodegradability (Couch et al., 2016). Nano-based “smart” and “active” food packagings confer several advantages over conventional packaging methods from providing better packaging material with improved mechanical strength, barrier properties, antimicrobial films to nanosensing for pathogen detection and alerting consumers to the safety status of food (Mihindukulasuriya and Lim, 2014).

**FIGURE 1:**
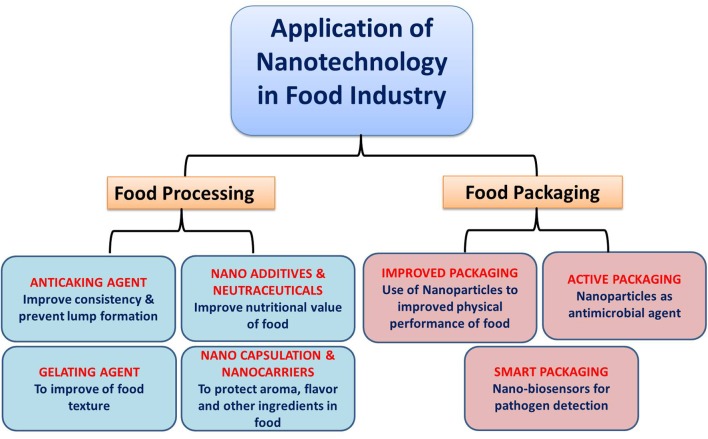
Schematic diagram showing role of nanotechnology in different aspects of food sectors.

Application of nanocomposites as an active material for packaging and material coating can also be used to improve food packaging (Pinto et al., 2013). Many researchers were interested in studying the antimicrobial properties of organic compounds like essential oils, organic acids, and bacteriocins (Gálvez et al., 2007; Schirmer et al., 2009) and their use in polymeric matrices as antimicrobial packaging. However, these compounds do not fit into the many food processing steps which require high temperatures and pressures as they are highly sensitive to these physical conditions. Using inorganic nanoparticles, a strong antibacterial activity can be achieved in low concentrations and more stability in extreme conditions. Therefore, in recent years, it has been a great interest of using these nanoparticles in antimicrobial food packaging. An antimicrobial packaging is actually a form of active packaging which contacts with the food product or the headspace inside to inhibit or retard the microbial growth that may be present on food surfaces (Soares et al., 2009). Many nanoparticles such as silver, copper, chitosan, and metal oxide nanoparticles like titanium oxide or zinc oxide have been reported to have antibacterial property (Bradley et al., 2011; Tan et al., 2013; **Figure 1**).

The application of nanoparticles is not limited to antimicrobial food packaging but nanocomposite and nanolaminates have been actively used in food packaging to provide a barrier from extreme thermal and mechanical shock extending food shelf-life. In this way, the incorporation of nanoparticles into packaging materials offers quality food with longer shelf-life. The purpose of creating polymer composites is to have more mechanical and thermostable packing materials. Many inorganic or organic fillers are being used in order to achieve improved polymer composites. The incorporation of nanoparticles in polymers has allowed developing more resist packaging material with cost effectiveness (Sorrentino et al., 2007). Use of inert nanoscale fillers such as clay and silicate nanoplatelets, silica (SiO_2_) nanoparticles, chitin or chitosan into the polymer matrix renders it lighter, stronger, fire resistance, and better thermal properties (Duncan, 2011; Othman, 2014). Antimicrobial nanocomposite films which are prepared by impregnating the fillers (having at least one dimension in the nanometric range or nanoparticles) into the polymers offer two-way benefit because of their structural integrity and barrier properties (Rhim and Ng, 2007).

## Nanosensors for Pathogen Detection

Nanomaterials for use in the construction of biosensors offers the high level of sensitivity and other novel attributes. In food microbiology, nanosensors or nanobiosensors are used for the detection of pathogens in processing plants or in food material, quantification of available food constituents, alerting consumers and distributors on the safety status of food (Cheng et al., 2006; Helmke and Minerick, 2006). The nanosensor works as an indicator that responds to changes in environmental conditions such as humidity or temperature in storage rooms, microbial contamination, or products degradation (Bouwmeester et al., 2009). Various nanostructures like thin films, nanorods, nanoparticles and nanofibers have been examined to their possible applications in biosensors (Jianrong et al., 2004). Thin film-based optical immunosensors for detection of microbial substances or cells have led to the rapid and highly sensitive detection systems. In these immunosensors, specific antibodies, antigens, or protein molecules are immobilized on thin nano-films or sensor chips which emit signals on detection of target molecules (Subramanian, 2006). A dimethylsiloxane microfluidic immunosensor integrated with specific antibody immobilized on an alumina nanoporous membrane was developed for rapid detection of foodborne pathogens *Escherichia coli* O157:H7 and *Staphylococcus aureus* with electrochemical impedance spectrum (Tan et al., 2011). Nanotechnology can also assist in the detection of pesticides (Liu et al., 2008), pathogens (Inbaraj and Chen, 2015), and toxins (Palchetti and Mascini, 2008) serving in the food quality tracking–tracing–monitoring chain.

Biosensors based on carbon nanotubes also gained much attention due to their rapid detection, simplicity and cost effectiveness and have also been successfully applied for the detection of microorganisms, toxins, and other degraded products in food and beverages (Nachay, 2007). Toxin antibodies attached to these nanotubes causes a detectable change in conductivity when bound to waterborne toxins and therefore are used to detect waterborne toxins (Wang et al., 2009). Further, the use of electronic tongue or nose which is consists of the array of nanosensors monitor the food condition by giving signals on aroma or gases released by food items (Garcia et al., 2006). The quartz crystal microbalance (QCM)-based electric nose can detect the interaction between various odorants and chemicals that have been coated on the crystal surface of the QCM. Many studies on small molecule detection have used quartz crystal surfaces that have been modified with different functional groups or biological molecules, such as amines, enzymes, lipids, and various polymers (Kanazawa and Cho, 2009).

## Safety Issues

Besides a lot of advantages of nanotechnology to the food industry, safety issues associated with the nanomaterial cannot be neglected. Many researchers discussed safety concerns associated with nanomaterial giving emphasis on the possibility of nanoparticles migrate from the packaging material into the food and their impact on consumer’s health (Bradley et al., 2011; Jain et al., 2016). Although a material is being considered as GRAS (generally regarded as safe) substance, additional studies must be acquired to examine the risk of its nano counterparts because the physiochemical properties in nanostates are completely different from that are in macrostate. Moreover, the small size of these nanomaterials may increase the risk for bioaccumulation within body organs and tissues (Savolainen et al., 2010). For example, silica nanoparticles which are used as anti-caking agents can be cytotoxic in human lung cells when subjected to exposure (Athinarayanan et al., 2014). There are a lot of factors that affect dissolution including surface morphology of the particles, concentration, surface energy, aggregation, and adsorption. A model to study the migration of particles from food packaging has been developed by Cushen et al. (2014). They studied the migration of silver and copper from nanocomposites and observed that the percentage of nanofiller in the nanocomposites was one of the most crucial parameters driving migration, more so than particle size, temperature, or contact time. Since every nanomaterial has its individual property, therefore, toxicity will likely be established on a case-by-case basis (Mahler et al., 2012). Further, regulatory authorities must develop some standards for commercial products to ensure product quality, health and safety, and environmental regulations.

## Conclusion

Over past years the popularity of the uses of structures on the nanometer scale in the food sector is increasing, therefore, interest and activities in this research area have greatly focused. As nanobiotechnology steps forward, devices or material based on this technology become smaller and more sensitive. Its applicability in the areas of food packaging and food safety are well known. Additionally, promising results have been achieved in food preservation using nanomaterial where they might protect the food from moisture, lipids, gases, off-flavors, and odors. They offer excellent vehicle systems to deliver bioactive compounds to the target tissues. Although the advances in nanotechnology are paving new paths day by day, there still persist many challenges and opportunities to improve the current technology and also issues about the consequences of nanotechnology that must need to be addressed in order to alleviate consumer concerns. The transparency of safety issues and environmental impact should be the priority while dealing with the development of nanotechnology in food systems and therefore compulsory testing of nano foods is required before they are released to the market.

## Author Contributions

TS and SS designed, conceived, and wrote the manuscript. PK helped in writing and editing. VW, VB, and IR critically reviewed, edited, and finalized the manuscript for submission.

## Conflict of Interest Statement

The authors declare that the research was conducted in the absence of any commercial or financial relationships that could be construed as a potential conflict of interest.
