# Corrigendum: Application of Nanotechnology in Food Science: Perception and Overview

**DOI:** 10.3389/fmicb.2017.02517

**Published:** 2017-12-12

**Authors:** Trepti Sing, Shruti Shukla, Pradeep Kumar, Verinder Wahla, Vivek K. Bajpai, Irfan A. Rather

**Affiliations:** ^1^Department of Microbiology, Gurukula Kangri University, Haridwar, India; ^2^Department of Energy and Materials Engineering, Dongguk University-Seoul, Seoul, South Korea; ^3^Department of Forestry, North Eastern Regional Institute of Science and Technology, Itanagar, India; ^4^Department of Applied Microbiology and Biotechnology, Yeungnam University, Gyeongsan-si, South Korea

**Keywords:** nanoparticles, food safety, food preservation, functional food, food nutrition, nano-processed food product

Irfan A. Rather was not included as an author in the published article. The authors apologize for this error and state that this does not change the scientific conclusions of the article in any way.

The original article has been updated.

## Author contributions

TS and SS designed, conceived, and wrote the manuscript. PK helped in writing and editing. VW, VB, and IR critically reviewed, edited, and finalized the manuscript for submission.

### Conflict of interest statement

The authors declare that the research was conducted in the absence of any commercial or financial relationships that could be construed as a potential conflict of interest.

